# The anti-virulence effect of cranberry active compound proanthocyanins (PACs) on expression of genes in the third-generation cephalosporin-resistant *Escherichia coli* CTX-M-15 associated with urinary tract infection

**DOI:** 10.1186/s13756-019-0637-9

**Published:** 2019-11-20

**Authors:** Shivanthi Samarasinghe, Ruth Reid, Majid AL-Bayati

**Affiliations:** Molecular Microbiology, Leicester School of Allied Health Sciences, Faculty of Health & Life Sciences, The Gateway, De Montfort University, Leicester, LE1 9BH UK

**Keywords:** Urinary tract infections; virulence, Uropathogenic *Escherichia coli*, UPEC, *E. coli* CTXM-15, Cranberry, Proanthocyanins (PACs)

## Abstract

**Background:**

Urinary tract infections (UTIs) are one of the most common infections found in humans, with uropathogenic *Escherichia coli* (UPEC) being the most common cause. Prevention of UTI is a major global concern due to its recurrent nature, medical cost, and most importantly, the increased antimicrobial resistance among UPEC. The resistance in UPEC is mainly due to the Extended-Spectrum β-lactamases (ESBL), particularly the *E. coli* CTXM-15 type which is known for its rapid dissemination worldwide. Treatment options for *E. coli* CTXM-15 have become limited over recent years because of their multi-drug resistance, hence anti-virulent strategies based on herbal remedies, have considered as a viable option. The cranberry product, Cysticlean® capsules, contain 240 mg of proanthocyanins (PACs), which have been shown to significantly inhibit *E. coli* adherence, both in vitro and ex vivo, to uroepithelial cells.

**Method:**

In this study, the cephalosporin-resistant *E. coli* isolate NCTC 1553 (*E. coli* CTXM-15) was analysed by qRT-PCR (quantitative Reverse Transcriptase -Polymerase Chain Reaction) for the expression of virulence factors after treatment with Cysticlean®. qRT-PCR was carried out to detect virulence determinants encoding for toxins SAT, and USP, the iron acquisition system ChuA, the protectins SoxS, KPSM, TraT and RecA, the antibiotic resistance gene CTX-M (encode β-lactamases), and the transporters IdfB and HcaT.

**Results:**

Cysticlean® significantly reduced the expression of all ten selected genes encoding for virulence factors and β-lactamases.

**Conclusion:**

Cranberry product Cysticlean® could represent a practicable alternative option for the prevention of recurrent UTI caused by multi-drug resistant *E. coli* CTXM-15, as the product acts on multiple bacterial targets.

## Introduction

Urinary tract infections (UTIs) are among the most common infectious disease affecting humans and have been estimated to occur in at least 50% of women at some stage during their lives. They are responsible for more than 7 million visits to physicians annually and 15% of all prescribed antibiotics in the US and European countries [1]. Because of the highly prevalent nature of UTIs, they represent a public health and economic burden. In the United States, in 2010, the annual direct and indirect cost of UTIs was estimated at $2.3 billion [2], and in the UK it has been estimated that the NHS has spent £434 million in 2013/14 to treat UTIs [3]; similarly, many other countries also incurred high medical costs in dealing with this common infection [2].

UTI are caused mainly by Uropathogenic *E. coli* (UPEC), which accounts for approximately 80% of all UTIs [4]. Common symptoms include painful, frequent and urgent need to urinate and can lead to irreversible kidney damage and even death [5]. Adjunctive treatment of UTI and prevention of recurrent UTI has become a global concern due to associated morbidity and increased medical cost associated with this infection [6]. UPEC associated with high levels of ESBL gene carriage [7] and the CTX-M β-lactamase family is the predominant ESBL type in many countries. Within this family, CTX-M-15 considered the most prevalent genotype worldwide [8]. It was demonstrated that CTX-M-15 is associated with an extensive pattern of antimicrobial resistance to a large number of antibiotics such as penicillins, cephalosporins, monobactams, and carbapenem [9]. Moreover, CTX-M-producing UPEC also exhibits coresistance to other large antimicrobial families such as aminoglycosides and fluoroquinolones [9]. Hence, this increasing level of UPEC multi-drug resistance is a great health and economic concern as it limits the therapeutic options available for the treatment of common bacterial infections such as UTIs.

In 2018, the World Health Organisation (WHO) composed a global priority list of antibiotic resistant bacteria and categorised the third-generation cephalosporin-resistant *Enterobacteriaceae*, as a global priority one group of pathogens, that needed urgent research and development of novel antibiotics [10]. CTX-M-producing UPEC belongs to the third-generation cephalosporin-resistant *Enterobacteriaceae* family, highlighted the importance of development of novel antibiotic for UTI associated with this pathogen. As a part of the therapeutic strategies designed to combat antimicrobial resistance among the UTI pathogens, the use of alternative methods such as anti-virulence strategies, which manipulates virulence factors of the bacteria rather than bacterial growth factors, are considered an effective tool as they have a lower risk of resistance development [11]. It is thought that the main reason for UPEC being so successful is their expression of a wide range of virulence factors [4]. Virulence genes are commonly located on mobile genetic elements such as pathogenicity islands, transposons, bacteriophages or plasmids and are generally spread by DNA horizontal transfer [12]. Understanding the role of virulence factors and their expression enables research that aims to discover novel therapeutic mechanisms that can limit bacterial infection. Virulence factors important to UPEC infection of the urinary tract include adhesins, capsules, toxins and iron-acquisition systems [12]. These virulence factors contribute to the colonization and invasion of the urinary tract, leading to biofilm formation, tissue damage and ascension to the bladder and kidneys. Research suggests that adhesins, or fimbriae, are the most important determinant of virulence in the infection of the urinary tract and have therefore been studied extensively.

It has been demonstrated that cranberries can decrease the virulence of UPEC, however little work has been completed on virulence factors other than for adhesins. Many in vivo and in vitro results have shown a significant inhibition of the adherence of *E. coli* to uroepithelial cells [13, 14]. A significant improvement in symptoms has been seen, including the reduction in painful, frequent and urgent urination [15]. Most of the above studies were focused on considering the cranberries as a whole fruit than its active compound, PACs, hence for this study the cranberry capsule Cysticlean® was chosen, which contains 240 mg of PACs. This study focusses on the toxins SAT, and USP, the iron acquisition system ChuA, the protectins SoxS, KPSM, TraT and RecA, the antibiotic resistance gene CTX-M and the genes responsible for the normal function of the cell, IdfB and HcaT. In this study, the CTX-M producing *E. coli* isolate NCTC 1553 was analysed via qRT-PCR for the expression of virulence factors, both before and after treatment with the cranberry product Cysticlean®. The results reported herein show that cranberry treatment reduces the expression of multiple virulence factors. These data suggest that cranberry’s mechanism may be multifaceted.

## Materials and methods

### Bacterial isolate and cranberry capsules

*Escherichia coli* isolate NCTC 13353 (anti-microbial resistance resistant reference strain from Public Health England, UK) expressing the resistance gene CTX-M-15 was used in all parts of this study. Cysticlean® capsules were purchased from VITA GREEN Europe S. A, which contained 240 mg of PACs per capsule.

### Minimum inhibitory concentration

In order to determine the concentration of cranberry required for use in the RT-qPCR experiment, the minimum inhibitory concentration was determined using the broth macro dilution method in triplicates. A 60 mg/ml PAC stock solution in 20 ml minimal medium (M9 Minimal Salts, 5X, Sigma Aldrich, Dorset, UK) was used for serial dilutions of the following concentrations of Cysticlean®: 30, 15, 7.5, 3.75, 1.88, 0.94, 0.47, 0.23, 0.12, 0.059, 0.029 mg/ml. An overnight culture (10^8^ CFU/ml) of *E. coli* NCTC 1553 was diluted to 0.5 McFarland standard in minimal medium, and 100 μl of this diluted culture was added to each concentration. Cultures were incubated at 37 °C with shaking overnight. The MIC was determined as the lowest concentration with no visible growth. A positive control, consisting of minimal medium and *E. coli*, and a negative control, consisting of minimal medium only, were included.

### RNA extraction

A 10 ml volume of the MIC concentration was prepared in minimal medium. An overnight culture of *E. coli* NCTC 1553 was diluted to 0.5 McFarland standard, 100 μl of which was added to the cranberry solution and incubated with shaking at 37 °C for 4 h. A second untreated culture was also incubated as an untreated control. A negative control consisting of minimal medium only was also included. All cultures were then filtered with a Whatman 1 and 0.45 membrane (GE Healthcare Life Sciences, Buckinghamshire, UK) before RNA lysate was extracted using the Cells-to-CT™ 1-Step TaqMan™ Kit (ThermoFisher Scientific, Loughborough, UK) following the manufacturer’s instructions, with the exception that the lysis + DNase time was doubled to 10 min. RNA concentration was measured using a Qubit 3 fluorometer (ThermoFisher Scientific, Paisley, UK). The quality of RNA and the presence of any DNA was assessed using agarose gel electrophoresis.

### Design of primers and probes for reverse transcriptase qPCR (RT-qPCR)

Primers and Taqman probes were designed using the PrimerQuest Tool (Integrated DNA Technologies) using the custom design parameters listed in Table 1. Primers were designed to be 20–24 bp, with a melting temperature of approximately 60 °C. The GC content was designed to be 40–60. Taqman probes were designed to be 20–30 bp in length, with a melting temperature of 7–10 °C higher than that of the primers, and with a GC content of 35–65%. The OligoAnalyzer 3.1 (Integrated DNA Technologies Inc., Illinois, USA, https://eu.idtdna.com/calc/analyzer) was used to identify and possibility of any secondary structure and primer dimers. Whilst non-specific products do not produce results in Taqman qPCR, and were avoided because they reduce the efficiency of the reaction. The FAM fluorophore was added to the 5′ end of the probe and the Black Hole Quencher®-1 was added to the 3′ end using the custom DNA oligo tool (Integrated DNA Technologies). Probes were ordered on a 100 nm scale and purified by high performance liquid chromatography (HPLC). Primers were ordered on a 25 nm scale by standard methods. Both primers and probes were ordered as “lab ready”, 100 μM in IDTE, PH 8.0.

**Table 1:** Sequences of primers and probes used in this study

	Forward	Reverse	Probe	Amplicon size (bp)	Reference
*16 s*	GGAGACTGCCAGTGATAAA	GAGGTCGCTTCTCTTTGT	CCTTACGACCAGGGCTACACACGT	113	This study
*kpsM*	GTTATATGGGACCGTCATATT	GCCACACTCTTTCGTATC	CCCAGAGCTTGCGGATTATCAATC	125	This study
*chuA*	CGATGGGCGAAATGTATAA	GTTAGTTTCCGGACGTAAG	TCTCGATTGGTCGCTTCTATACCA	98	This study
*CTX-M*	GAAAGCGAACCGAATCT	GACATCGTCCCATTGAC	ACCTTGTTAACTATAATCCGATTGCGG	101	This study
*sat*	AGAAATATGGCATCTGTCACC	CAGACGATATAGTCGGTGTTTC	CAGCAACCATTACTCTGGGACAGC	97	This study
*usp*	TGAGTTCTGGTATGAGGAAGA	CCCGTATGAACACCATACAC	TACTGCCTGCTAGTGCTTTCTGCC	126	This study
*soxS*	AGGGTTGATTACCAGTATCC	AACATATCGCAACACATCAC	ACAGCGGCAATCAGCGGCGATATA	70	This study
*recA*	GAGACAATTTGGTACTCCATC	GATATCCAGTGAAAGCGAAC	CGCCTGGGTGAAGACCGTTCCAT	90	This study
*traT*	AGATTGCAGAGCGTACTAAG	ACACGGGTCTGGTATTTATG	CAACGGATAATGTTGCCGCCCTGC	127	This study
*ihfB*	GACGGTTGAAGATGCAGTAA	CGGTAGTGCAAAGAGAAACT	AGCATATGGCCTCGACTCTTGCGC	108	This study
*hcaT*	GGCATTATGGGAGCAACTAC	CTGGCGAGCAGCAATATAAC	ACCACTATCAACCACGGCAACGCC	114	This study

Primer and probe sets were designed using the PrimerQuest Tool (Integrated DNA Technologies). All probes contained a FAM fluorophore at the 5′ end and a black hole quencher at the 3′ end.

### Primer specificity

**Fig. 1:**
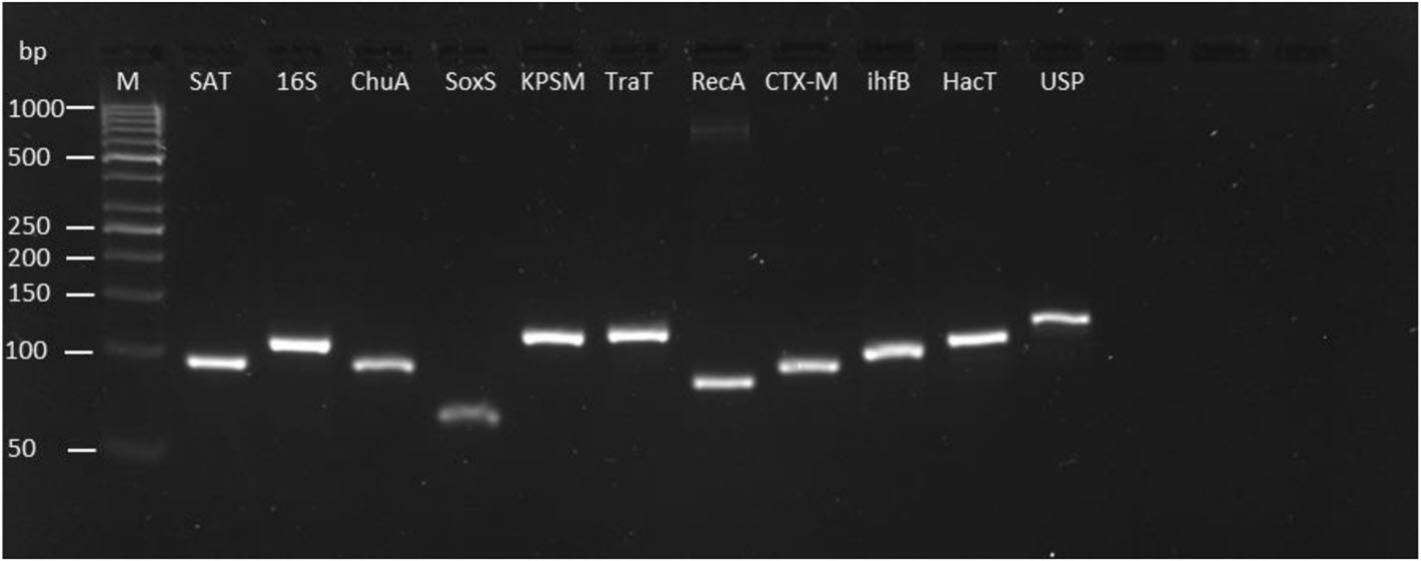
The specificity of each primer pair investigated by qRT-PCR for this study. End amplified products of qPCR were visualised using agarose gel electrophoresis. All genes were detected at the expected bp size and single band was observed for each primer pair indicating primer specificity. M, Molecular Weight marker, GeneRuler 50 bp DNA Ladder (Thermofisher, UK). Products and ladder ran on 4% Agarose gel.

RT-qPCR primers were checked for specificity by end-point PCR using extracted DNA, as presented in Fig. 1. Cycling parameters were as follows: initial denaturation at 95°C for 20 s, followed by 40 cycles of 95 °C for 15 s each and 60 °C for 1 min.

### Primer efficiencies

Primer efficiencies were calculated using serial dilutions of amplicons (diluted 1:1000) in triplicate and by plotting the resulting data to produce standard curves for each primer pair selected. The amplification efficiency = [10(−1/slope)] – 1. A correlation coefficient (R^2^) of 0.99 or greater was considered. An amplification efficiency of 80–120% was considered and the efficiencies of selected primers are presented in Table 2.

**Table 2:** The efficiencies of the primers used in the development of the RT-qPCR study

Primer	Efficiency %
*16S* (reference gene)	84.3
*recA*	111.1
*CTX-M*	99.7
*sat*	111.6
*traT*	94.3
*chuA*	115.6
*kpsM*	101.7
*soxS*	103.0
*usp*	90.2
*hcaT*	118.9
*IhfB*	115.3

Primer efficiencies were calculated using serial dilutions of amplicons 1:1000. An efficiency of 80–120% was considered.

### RT-qPCR method

The Cells-to-CT™ 1-Step TaqMan™ Kit was used for RT-qPCR. This kit allows for expression analysis from cultured cells without the requirement for extensive RNA purification. The mastermix contains a reverse transcriptase, allowing for a 1-step RT-qPCR. The Cells-to-CT™ mastermix contained the reverse transcriptase and allowed cDNA conversion during qPCR. qPCR reactions were carried out using the PikoReal® 96 well real-time PCR platform (ThermoFisher, Loughborough, UK). PCR amplification reactions were performed using three technical replicates, in a volume of 10 μl containing 2.5 μl TaqMan® 1-Step qRT-PCR Mix (initial concentration not known), with final concentrations of 500 nM primers, 250 nM probe and 1 μl of RNA lysate. Cycling parameters for the RT-qPCR assay were, an initial reverse transcription step at 50 °C for 5 min, reverse transcriptase inactivation/initial denaturation at 95 °C for 20 s, followed by 40 cycles of 95 °C for 15 s and, finally, 60 °C for 1 min. A no-reverse transcriptase control was implemented in triplicate by adding the mastermix containing reverse transcriptase after the reverse transcription step of the RT-qPCR assay. A no template control consisting of water instead of RNA was also added in triplicate. The experiment was performed twice (using two biological replicates) and the representative values presented were the means of the triplicates.

### Statistical analysis

Quantification cycle (Cq) values were obtained after completion of the RT-qPCR. The relative abundance of the target gene in each sample (treated) and calibrator (untreated) was calculated using the Cq value of the internal reference gene 16SrRNA as a control and the relative expression level of the target gene = 2^-ΔΔCq^ (where ΔCq = Cq_(target gene)_ - Cq_(reference gene)_, and ΔΔCq = ΔCq_(test)_ - ΔCq_(calibrator)_) as described previously [16], with a modification to account for primer efficiencies using Microsoft Excel. Primer efficiencies were calculated using serial dilutions of amplicons (diluted 1:1000), and this efficiency was also used in the ∆∆Ct calculations. All statistical analyses were carried out using the GraphPad Prism software (version 5; GraphPad Software Inc.; La Jolla, CA, USA). To determine the significant differences between treated and untreated samples, the t-test was performed. A difference with a *p*-value of <0.05 was considered statistically significant.

## Results

### Mic

The MIC for *E. coli* strain NCTC 13353 was determined to be 15 mg/ml, and this was used in all subsequent experiments.

### RT-qPCR

**Fig. 2:**
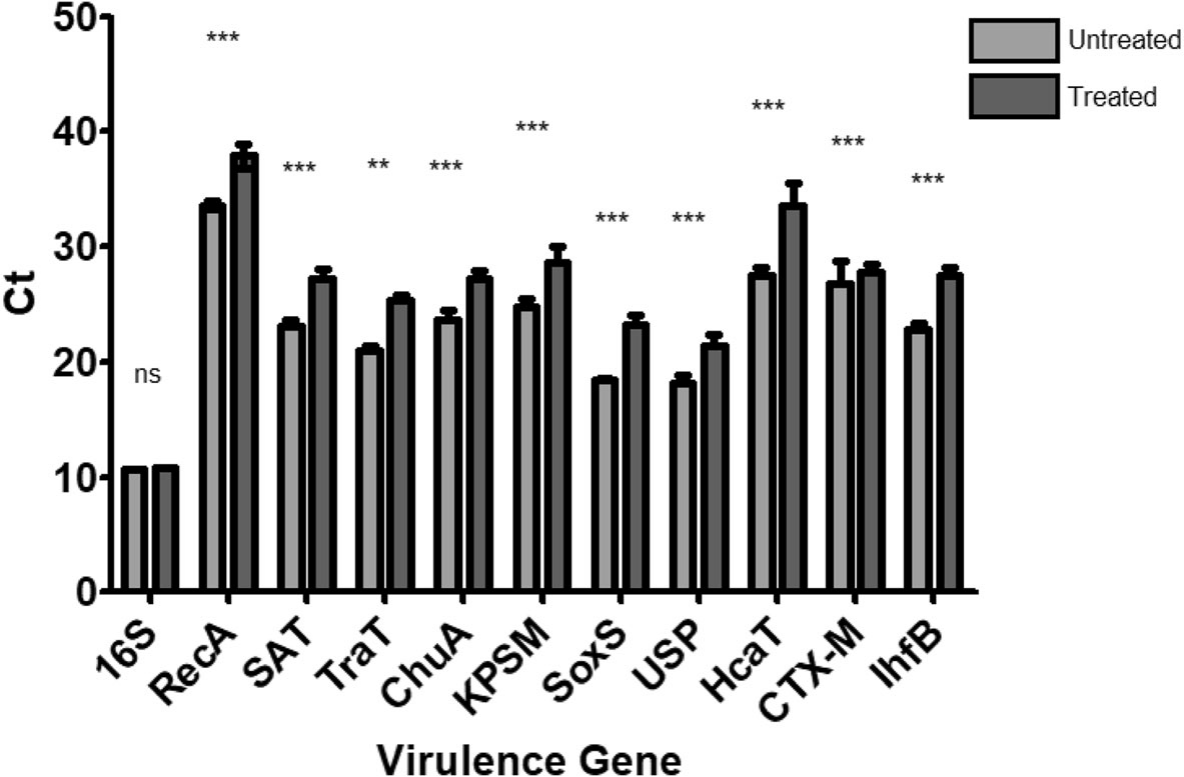
Ct values as a measure of expression of the selected virulence genes. Genes for toxin production, iron acquisition, antibiotic resistance protectins and normal function of the cell were tested after exposure to Cysticlean® using RT-qPCR all genes were significantly downregulated when tested using a t-test. * *p* = 0.01 to 0.05 ** *p* = 0.001 to 0.01 *** *p* = < 0.001 ns = not significant

**Fig. 3:**
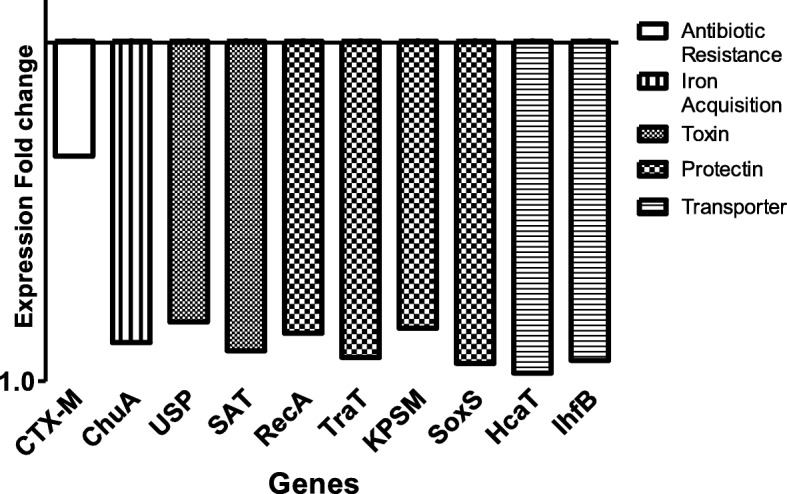
The expression fold change of genes antibiotic resistance, iron acquisition, toxins, protections and genes responsible for the normal function of the cell*.* The results of the expression fold change were normalized against the reference gene 16 s and then were taken away from one to show the genes were downregulated

In this study, RT-qPCR was used to evaluate the impact of exposure of *E. coli* NCTC 13353 cells to the commercial cranberry product Cysticlean® (at MIC; for 4 h) on the expression of ten genes that have been shown to be associated with the virulence of UPEC. The selected genes included the toxins SAT and USP, the iron acquisition system ChuA, the protectins SoxS, KPSM, TraT and RecA, the antibiotic resistance gene CTX-M, and the genes responsible for the normal function of the cell, IdfB and HcaT. Figure 2 shows the mean average Ct values as a measure of expression of these 10 genes in the absence or presence of cranberry treatment. There was a significant decrease (*P* < 0.05) in the Ct value for each gene in the presence of cranberry, indicating a decrease in expression. Figure 3 shows the decrease as a Double Delta Ct Value (ΔΔCt) -1. By presenting the data as (ΔΔCt) -1, a value of 0 would indicate no change while a value between 0 and 1 indicates a downregulation in expression. The t-test showed that all the genes tested were significantly downregulated (*P* < 0.05) following treatment with cranberry.

It is not possible to run melting curve analyses on Taqman assays due to the degenerative nature of Taqman probes. Non-specific products do not produce results in Taqman assays; therefore, it is not necessary to run products on an agarose gel; however, non-specific products should in any case be avoided because they reduce the efficiency of the reaction. Taqman assays were checked for specificity before running qPCR.

## Discussion

**Fig. 4:**
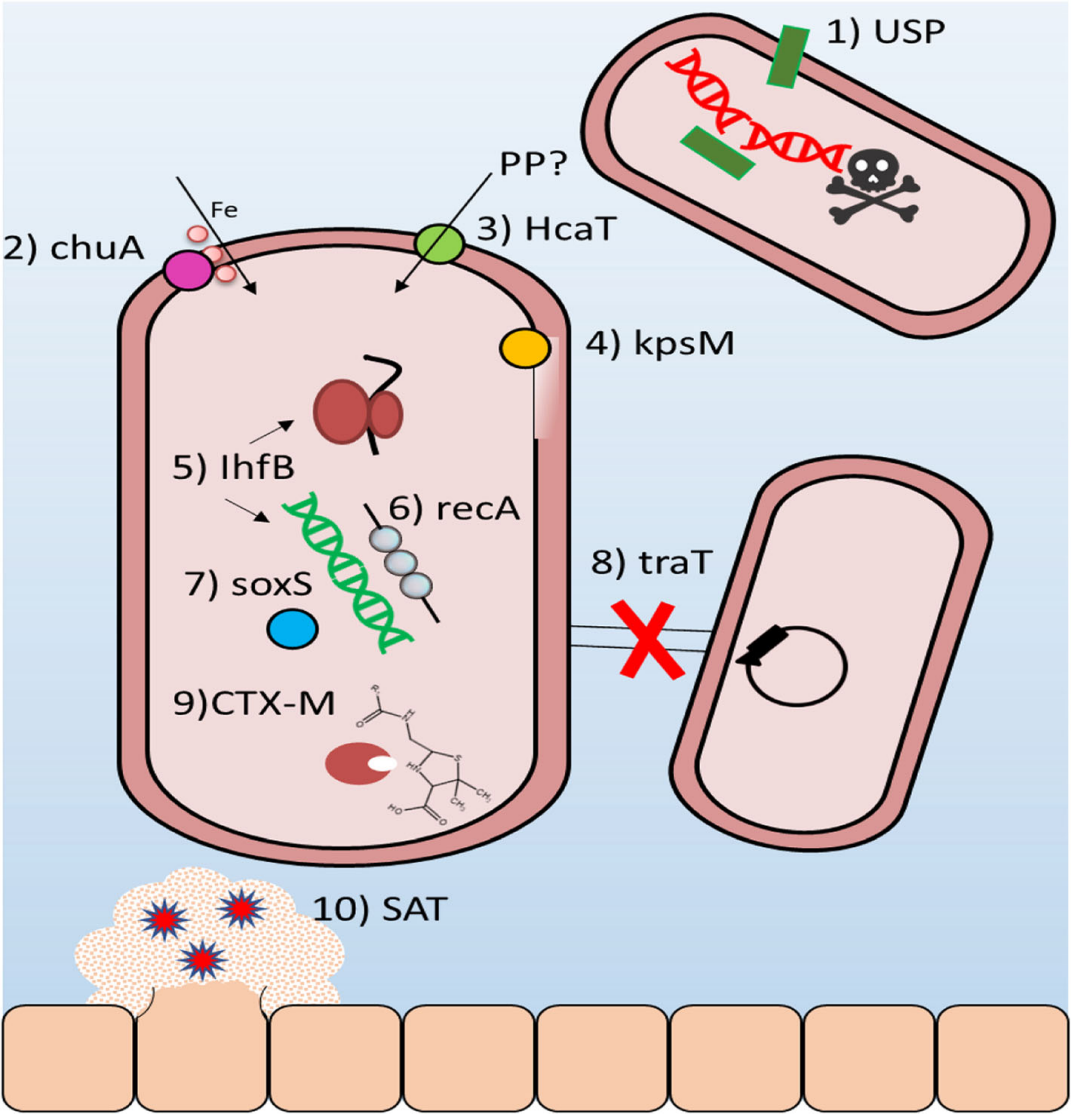
Reported mechanisms of action for 10 Virulence genes in *Escherichia coli* are downregulated by cranberry treatment. This study has shown that the following a selection of 10 genes, which encode for virulence factors with different mechanisms, were all downregulated when exposed for Cysticlean® for 4 h at MIC. 1) uropathogenic specific protein gene (*usp*) encode for bacteriocin that possesses DNase activity that can degrade plasmid DNA of competing uropathogens. 2) *chuA* encodes for outer membrane protein that plays a key role in heme uptake. 3) *hcaT* encodes for permease for the uptake of phenylpropionic acid (PP), an aromatic acid that can be utilised as a carbon and energy source. 4) *kpsM* encodes for protein that plays a role in group 2 capsular polysaccharide synthesis. 5) *ihfB* encodes for specific DNA-binding protein that functions in genetic recombination as well as in transcriptional and translational control. 6) *recA* encodes for protein that catalysise ATP-driven homologous pairing and strand exchange of DNA molecules necessary for DNA recombinational repair. 7) s*oxS* encode for a transcriptional activator of the superoxide response regulon of *E. coli*. 8) *traT* encodes for protein that prevents unproductive conjugation between bacteria carrying like plasmids. 9) *CTX-M* produces beta-lactamases that can render beta-lactams inactive. 10) *sat* encode for a secreted autotransporter toxin exhibits cytopathic activity and loosening of cellular junctions. These data suggested that cranberry’s antimicrobial action is multifaceted

Reports have shown that numerous genes are implicated in the virulence and fitness of UPEC, therefore downregulating the expression of these genes could add to the efficiency of antibiotic treatment and could reduce the likelihood of treatment failure [17]. In this study, the selected ten genes playing significant roles in the normal function of the cell, such as iron acquisition, toxin production and stress survival in the third-generation cephalosporin resistant *E. coli*, and their relative gene expression was determined after exposure to the cranberry product Cysticlean® using qRT-PCR. The results showed that Cysticlean® significantly reduces the expression of a wide variety of genes responsible for fitness and virulence. To the best of our knowledge, this is the first study to access the effect PACs, the active compound of the cranberry on the expression of virulence and antibiotic resistance determinants. A reported mechanism of action the selected genes is represented in Fig. 4.

Reports have suggested that USP functions as a bacteriocin, targeting competing bacterial strains (depicted in Fig. 4). It has also been shown to enhance infectivity in the urinary tract and play a role in UPEC pathogenesis [18, 19]. In a study by Bauer et al. [20], it was shown that the *usp* gene was found in 80% of cystitis isolates [20]. A reduction in the expression of *usp* could lead to this bacterium being outcompeted by other non-pathogenic bacteria and a reduction in its ability to cause pyelonephritis. It is especially advantageous for UPEC to be able to utilize haem and/or haemoglobin, as the urinary tract is naturally low in iron. UPEC secrete cytotoxins that can access the haem reservoir within cells. These two capacities combined could aid iron acquisition throughout the infection process [20, 21]. ChuA is an outer membrane receptor responsible for the transport of haem into the cell for utilization as iron (see Fig. 4) [22]. A reduction in iron transport into the cell could lead to reduced growth of the bacteria, or potentially even cell death. In a study, cranberry treatment resulted in the up-regulation of genes that encode iron metabolism regulators (*fur*), transport protein (*feoAB*), genes involved in the biogenesis pathway of Fe-S clusters (*iscAS*) and iron storage (*ftnA, acnA, sodB*) [13]. This is the first study to investigate the expression of the c*huA* gene after exposure to a cranberry product. Combining the literature with the results of this study, cranberry can be said to cause the overexpression of iron-acquisition genes, but a downregulation of haem membrane receptors. It has been suggested that the expression of c*huA* is influenced by RfaH in UPEC [22]. Therefore, it may be that Cysticlean® has an effect on RfaH that requires further investigation.

SAT is a serine protease which is found predominantly in uropathogenic strains of *E. coli*, and which has been shown to elicit a cytopathic effect on cultured epithelial cells (see Fig. 4); it has also been shown to be associated with pyelonephritis [13]. A reduction in the expression of *sat* gene could lead to a reduction in the ability of UPEC to cause pyelonephritis. KpsM is responsible for capsule production, protecting the bacterial cell from host defenses such as engulfment by phagocytes and the bactericidal effects produced by the complement system (see Fig. 4). KpsM is commonly associated with pyelonephritis [13]. A downregulation in the expression of *kpsM* gene could lead to cell death by phagocytes and the complement system.

SoxS increases tolerance to stress, as activated by cytotoxic weapons produced by the human immune system such as nitric oxide, and leads to direct activation of antibiotic resistance factors [23]. Reduction in the expression of *soxS* could lead to bacteria that are less tolerant to stress and generally less antibiotic resistant. In a study, cranberry treatment caused significant overexpression of genes involved in the stress response (*arcA, rpoS, cpxR*), periplasmic stress (*rseAC*) and oxidative stress (*oxyR*). This being said, they did not specifically report results on the *soxS* [23]. RecA plays a role in DNA recombination by catalysing the homologous pairing driven by ATP and the strand exchange of DNA molecules (Fig. 4) [24]. A reduction in recombinational repair could lead to bacteria that are less able to survive.

TraT is an outer membrane lipoprotein that is responsible for preventing unproductive conjugation between bacteria carrying like plasmids (Fig. 4). It has also been shown to interfere with complement-mediated killing, imparting a mild amount of serum resistance [21]. A downregulation in the expression of *traT* gene could lead to an increase in unproductive conjugation and a decrease in serum resistance. The h*caT* gene plays a role in the catabolism of 3-phenylpropionate and cinnamic acid, the products of which are fed into the mhp pathway (see Fig. 4-III). IhfB is responsible for the activation of transcription via sequence-specific DNA binding and for site-specific recombination. It is uncommon to see changes in expression of these genes; indeed, they have been recommended as suitable reference genes in transcriptional analysis of *E. coli* [25]*.* Even though it was the least downregulated gene, it could be stipulated that the most important downregulation was that of the *CTX-M* gene that causes extended-spectrum β-lactamase (ESBL) production. ESBL-producing UTIs are a major problem in both the community and the hospital. Hence the results indicated that the reduction in the expression of *CTX-M* gene could lead to the increased susceptibility of other known resistant isolates.

This study suggests that the effects of Cysticlean® are multifactorial, effecting many different pathways. Other studies have shown that exposure of UPEC to cranberry PACs or cranberry powder results in the downregulation of the flagellin gene *fliC,* which results in impeded motility via swimming and swarming. Honey has also been shown to have a similar effect on virulence genes, though its ability to reach the urinary tract intact has been questioned [26]*.*

Based on above literature and current findings, two complementary mechanisms have been suggested to explain the ability of the American cranberry to reduce urinary tract infections. The first mechanism has been credited to its biocidal activity, as enabled through the production of several elements that can damage bacteria. The second mechanism is attributed to its anti-virulence activity, through the downregulation and interference with multiple virulence factors associated with tolerance to stress and survival in the urinary tract. The latter could ultimately result in the weakening of bacterial survival abilities and its ability to cause infection [27]*.*

Recurrent UTI is commonly treated with antibiotic prophylaxis, which has been attributed to bacterial resistance*.* Antibiotic resistance is a major concern worldwide, therefore a re-evaluation of the treatment recommendations for UTI should be considered. An alternative to antibiotic prophylaxis could be treatment using cranberry products or a combination of cranberry and antibiotic treatment in an attempt to reduce resistance rates in UTIs.

Future studies into analysing the effects of the active compound PACs on other virulence and antibiotic resistance determinants and in other bacterial species that cause UTI need to be carried out. This should involve testing other virulence and antibiotic resistance genes; a microarray analysis may be more useful in this regard. Moreover, these analyses also be combined with phenotypic and in vivo studies to give a broad overview of the effects that cranberry treatment has. Other bacterial strains and species, as well those that are antibiotic resistant and non-resistant, that cause UTI should be studied to see if the effect of cranberry is broad-spectrum in nature. In this study, only one reference gene could be used. Multiple reference genes increase the accuracy of relative expression normalization and allow for appropriate quality control on the stability of the reference gene expression. Therefore, the use of one reference gene represents a particular limitation to this study.

## Conclusions

Taken together, the study showed that the cranberry active compound PACs has effective downregulation activity on the fitness, virulence and antibiotic resistance in the ESBL producing *E. coli* strain CTX-M 15, which is the most common bacterial pathogen associated with UTIs and that shows multi-drug-resistant abilities. Hence, the study results indicated the cranberry compound PACs may represent a promising anti-virulence strategy for the treatment and modulation of the UTIs caused by *E. coli* strain CTX-M 15. Based on the results of this study, PAC can be used as co adjunctive method to reduce antibiotic resistance rates in recurrent Urinary Tract Infection.

## Data Availability

Not applicable; Data sharing not applicable to this article as no datasets were generated or analyzed during the current study. Please contact author for data requests.
